# Whole Blood Gene Expression Profiles of Patients with a Past Aneurysmal Subarachnoid Hemorrhage

**DOI:** 10.1371/journal.pone.0139352

**Published:** 2015-10-06

**Authors:** Femke N. G. van ’t Hof, Ynte M. Ruigrok, Jelena Medic, Bahram Sanjabi, Pieter van der Vlies, Gabriel J. E. Rinkel, Jan H. Veldink

**Affiliations:** 1 Department of Neurology and Neurosurgery, Brain Center Rudolf Magnus, University Medical Center Utrecht, Utrecht, The Netherlands; 2 University of Groningen, University Medical Center Groningen, Department of Genetics, Groningen, The Netherlands; Heinrich-Heine University, GERMANY

## Abstract

**Background:**

The pathogenesis of development and rupture of intracranial aneurysms (IA) is largely unknown. Also, screening for IA to prevent aneurysmal subarachnoid hemorrhage (aSAH) is inefficient, as disease markers are lacking. We investigated gene expression profiles in blood of previous aSAH patients, who are still at risk for future IA, aiming to gain insight into the pathogenesis of IA and aSAH, and to make a first step towards improvement of aSAH risk prediction.

**Methods and Results:**

We collected peripheral blood of 119 patients with aSAH at least two years prior, and 118 controls. We determined gene expression profiles using Illumina HumanHT-12v4 BeadChips. After quality control, we divided the dataset in a discovery (2/3) and replication set (1/3), identified differentially expressed genes, and applied (co-)differential co-expression to identify disease-related gene networks. No genes with a significant (false-discovery rate <5%) differential expression were observed. We detected one gene network with significant differential co-expression, but did not find biologically meaningful gene networks related to a history of aSAH. Next, we applied prediction analysis of microarrays to find a gene set that optimally predicts absence or presence of a history of aSAH. We found no gene sets with a correct disease state prediction higher than 40%.

**Conclusions:**

No gene expression differences were present in blood of previous aSAH patients compared to controls, besides one differentially co-expressed gene network without a clear relevant biological function. Our findings suggest that gene expression profiles, as detected in blood of previous aSAH patients, do not reveal the pathogenesis of IA and aSAH, and cannot be used for aSAH risk prediction.

## Introduction

Subarachnoid hemorrhage (SAH) from a ruptured intracranial aneurysm (IA) is a severe subtype of stroke, occurring in relatively young people (mean age 50 years), of whom a third dies as a consequence of the aneurysmal SAH (aSAH).[1] It is known that both environmental exposures and genetic predisposition play a role in susceptibility of aSAH,[2] with an estimated heritability of around 40%.[3] The exact pathogenesis of IA development and subsequent aSAH is not exactly known, but processes like hemodynamic stress, matrix degeneration and inflammation appear to play a role.[4, 5]

Around 10% of the aSAH patients has one or more first degree relatives with aSAH, and unaffected first degree relatives are at increased risk of developing an aneurysms and having an aSAH.[6] IA are generally asymptomatic before rupture, and therefore have to be detected by screening. Magnetic resonance angiography (MRA) is currently the standard screening method for individuals at high risk for IA development and subsequent rupture, but screening has disadvantages in terms of costs and negative consequences.[7, 8] Moreover, screening is inefficient in first-degree relatives if only one relative is affected, although they have an increased life time risk of aSAH.[6, 9] Thus, we need tools to better detect persons with high risk of aneurysm development or rupture. Gene expression profiling in blood of previous aSAH patients may help to identify individuals who are at high risk for aSAH. Given the high long-term risk for developing new aneurysms in previous aSAH patients, aSAH seems to be a continuous disease of the vessel wall, making these patients suitable subjects for studying ongoing pathophysiologic processes involved in IA.[10]

Therefore, in this study, we compared gene expression profiles in blood between individuals who had survived an episode of aSAH and healthy controls. We aimed to gain more insight into the pathogenesis of IA and aSAH, and also to see whether these gene expression profiles can improve identification of individuals with an increased risk of aSAH.

## Materials and Methods

### Study design and subjects

Between August 2010 and January 2011, we sampled blood from 119 persons who had been treated for aSAH in the University Medical Center Utrecht (UMCU), the Netherlands. All patients visited the outpatient clinic at the UMCU for blood sampling. We included only patients who had the last episode of aSAH at least two years (median 7.5 years; range 2–23 years) before the blood sample collection to minimize the chance of detecting direct effects of the bleeding on gene expression profiles. Aneurysmal SAH was defined by symptoms indicative of SAH combined with subarachnoid blood on a computed tomography (CT) scan and a proven aneurysm at angiography (conventional angiogram, CT- or magnetic resonance (MR)-angiogram). Ruptured IA were treated by operative clipping or by coiling. In a subgroup of 16 patients, one or multiple unruptured IA were found in addition to the ruptured IA. Of these, five patients had an IA that was left untreated. The controls were genetically unrelated individuals accompanying the patient to the outpatient clinic (mostly spouses of the aSAH patients). When such unrelated individuals were unavailable, spouses of other patients visiting the neurology outpatient clinic served as controls. In total, we included 118 controls. For all participants, we obtained information about age, smoking history, hypertension (defined as a self-reported history of hypertension and/or use of antihypertensive medication) and presence of familial IA (defined as having one or more first-degree relative(s) with SAH or IA). All controls confirmed a negative history of SAH or IA.

The study was approved by the Medical Ethics Committee at the University Medical Center Utrecht, and all participants provided written informed consent.

### Blood sample collection and processing

Blood samples were obtained in the morning after overnight fasting. In each participant (i.e. cases and controls), we collected two PAXgene tubes (Qiagen) for genome-wide gene expression, and an EDTA tube for measurement of leukocyte differential counts. PAXgene tubes were frozen at -20°C after two hours at room temperature, until RNA was isolated using PAXgene extraction kits (Qiagen).

We excluded five cases and three controls with low RNA quality or quantity, defined as an RNA integrity number (RIN) value below 6. We also excluded one case with low RNA quantity after RNA amplification, defined as a 260/280 ratio below 1.8, measured using nanodrop (www.nanodrop.com). The remaining samples were hybridized to Illumina HumanHT-12v4 Expression BeadChips.

### Data quality control

R version 2.15.2 was used for quality control and statistical analysis of gene expression data.[11]

After calculating principal components (PCs), we excluded nine samples identified as outliers based on visual inspection of PC plots (S1 Fig). Seven samples showing inconsistency between reported gender and expression data based on at least two out of eight non-pseudoautosomal sex chromosome transcripts, and two duplicate samples (>99% gene expression correlation, measured using Pearson’s correlation coefficient) were excluded. In total, ten cases and eight controls were excluded after quality control.

All probe sequences were aligned to the NCBI build 36 reference genome using UCSC’s Genome Browser function BLAT.[12] We removed non-specific probes, defined as no or multiple hits with a sequence homology >95%, and non-autosomal probes (n = 13 188). Probes mapping to transcripts designated as ‘retired’ according to RefSeq (updated on 27 September 2010) and UniGene (build #228, release data 29 October 2010) databases were also excluded.

After exclusion of sample and probe outliers, the raw dataset was again quantile normalized and log_2_ transformed before further analyses.

### Data analysis

After exclusion of samples that did not surpass the quality control, we divided the remaining samples in a discovery set (2/3 of the dataset) and a replication set (1/3 of the dataset), with an equal distribution of cases and controls in each set. We performed four different analyses to investigate the gene expression profiles. Analyses 1 to 3 were aimed at gaining more insight in the pathogenesis of IA and aSAH and analysis 4 at exploring the possible use of these profiles in prediction of aSAH risk.

#### 1. Differential expression

We calculated case-control differences in expression of all probes in the discovery set, using logistic regression. To eliminate expression heterogeneity caused by known and unknown technical and biological background, data were normalized applying surrogate variable analysis (SVA). This produces surrogate variables for which expression levels can be corrected by calculating residuals in a linear regression model. This reduces batch specific background noise thereby increasing the ability to detect biologically meaningful signals.[13] As covariables, we included the 14 variables as specified by the SVA procedure. To correct for multiple testing, we calculated Benjamini Hochberg false discovery rates (FDR). All probes with a FDR-corrected p-value (p_FDR_) below 0.05 were tested for case-control difference in the replication set. Probes with p_FDR_ < 0.05 in the replication set were marked as significant.

Next, we created a list of 69 genes with a previously described association with IA (S1 Table), consisting of all genes in significant loci from previous genome-wide association studies (GWAS),[14–16] and genes associated with IA in at least three gene expression studies in IA tissue.[17] We checked the p-value from the differential expression analysis for probes mapping to these genes. Probes were significant if p-values were below 0.05 after Bonferroni correction for the number of tested probes.

#### 2. Co-differential co-expression (CDC)

Differentially expressed genes can be interacting with other genes to generate their effect on diseases. We tried to identify a differential gene regulating network involved in IA, based on co-differential co-expression.[18, 19] Therefore, we investigated which genes have a similar pattern of differential expression in IA cases and controls. First, we calculated residuals of gene expression levels after adjustment for known risk factors for SAH: age, sex, hypertension, smoking (ever or never) and familial IA. Leukocyte differential counts were not included as co-variables in the CDC analysis, because no significant differences (threshold p<0.05 using two-sample Wilcoxon test) between cases and controls were observed (S2 Table).

For the co-expression analyses, we used Weighted Gene Co-expression Network Analysis.[20] An adjacency matrix was defined between all genes under study, based on pair-wise correlations in all subjects in the discovery set. We used the Spearman rank correlations, to avoid the leverage of influential outliers.[21] On this co-expression matrix, standard hierarchical clustering with average linkage was applied, followed by gene group extraction from the resulting dendrogram, using a fixed cut height (0.96).[21] We created modules of co-expression based on the adjacency matrix, and for each gene we matched the significance level of differential expression to the module assignment. Next, we calculated the average gene significance level in each module based on analysis 1, and tested whether this level was significantly higher than expected by chance (p<0.05).

To test the reproducibility of these significant modules, we calculated preservation statistics for each module in the replication set, as described previously.[22] Specifically, the Z-summary statistic was investigated. This statistic captures the density and connectivity statistics, and adjusts for module size. DC modules with a Z-summary statistic > 10 (threshold for strong preservation evidence[22]) were considered as significantly preserved.

#### 3. Differential co-expression (DC)[21, 23]

Standard differential expression mainly investigates regulatory genetic variation that leads to expression level changes between cases and controls. Known disease genes, however, are often not differentially expressed because mutations in the coding region can affect the interaction of the gene with other genes, which will affect the co-expression pattern of the gene. Networks from gene expression data can be inferred by calculating all pair-wise correlations of the genes in a diseased and a control state and compare these networks based on differential co-expression.[21]

First, we created an adjacency matrix as described in paragraph 2 (‘Co-differential co-expression’), but this time by investigating the cases and controls separately, instead of all subjects in the discovery set together. Then the co-expression changes were computed from the difference in adjacency matrices. Modules of differentially co-expressed genes were extracted from this matrix, and these modules were randomly color-labeled. The statistical significance of differential co-expression of gene groups was assessed using 10 000 permutations of the data to generate a null distribution of the dispersion statistic, followed by a Bonferroni correction of the empirical p-values.[21] The dispersion statistic (*D*_*s*_) is a measure of correlation change for groups of genes.[24]

We selected modules with a Bonferroni-corrected p-value (p_Bonf_) below 0.05 for preservation testing in the replication set as described above. Next, we created correlation heatmaps of differentially co-expressed gene modules for the discovery set and replication set separately, using hierarchical cluster analysis based on gene correlation values.

**Biological relevance of gene modules:** We investigated the biological meaning of preserved modules based on (co-)differential co-expression in two ways. First, we tested the enrichment of genes involved in biological pathways in each module, using the database for annotation, visualization and integrated discovery (DAVID).[24] Biological pathways with p_Bonf_ < 0.05 were considered as significantly enriched. Second, we determined hub nodes in each module. Hub nodes or “hubs” take a central position in a network. They can be easily reached by most of the nodes of the network due to their central position.[25, 26] Several metrics can be used to identify hubs. Hub nodes generally display an above average high number of connections to other nodes in the network, a high level of (betweenness) centrality, a short average distance towards the other nodes of the network, and a low clustering coefficient.[25, 26] We identified hubs by computing a level of “hubness” for each node determining whether a node belonged to: (1) the top 20% of nodes showing the highest level of connectivity; (2) the top 20% of nodes showing the highest level centrality; (3) the top 20% of nodes showing the lowest path length; and/or (4) the top 20% nodes showing the lowest clustering coefficient.[27] Each node was assigned a score between 0 and 4, determined by the total number of hub criteria fulfilled. Regions showing a hub-score of 2 or higher were marked as hub nodes.

We looked up the function and published disease associations for the most important hub nodes at the website of the National Center of Biotechnology Information (http://www.ncbi.nlm.nih.gov). Next, we investigated whether there was any overlap between these hub genes and the list of 69 genes with a previously described association with IA.

#### 4. Prediction analysis of microarrays (PAM)

With this analysis, which uses the nearest shrunken centroid method,[28] we aimed to find the smallest set of genes in our data that can accurately classify samples as cases or controls. We computed a nearest shrunken centroid classifier for the SVA-normalized discovery dataset, and determined the amount of shrinkage by cross-validation. Next, we estimated FDRs for this classifier in the replication set. The optimal classifier gene set was determined by calculating sample misclassification errors for gene sets at different shrinkage thresholds.

## Results

A total number of 210 samples (103 cases and 107 controls) and 34 135 probes were included after quality control. Table 1 shows the baseline characteristics of the study population.

**Table 1:** Baseline characteristics of the study population.

Characteristics	Cases	Controls
Total number	103	107
Mean age (range)	60 (43–87)	60 (20–85)
Women *N* (%)	86 (83)	43 (40)
FIA *N* (%)	4 (4)	0 (0)
History of smoking *N* (%)	85 (83)	83 (78)
Hypertension *N* (%)	63 (61)	30 (28)
Mean time from SAH to study (range)	8 (2–23)	NA
Cases with additional aneurysms *N* (%)	14 (14)	NA

FIA indicates familial intracranial aneurysm, N: number, SAH: subarachnoid hemorrhage, NA: not applicable.

### 1. Differential expression

We observed no probes with a significant (p_FDR_ < 0.05) case-control difference in expression in the discovery set. Consequently, no probes were selected for further analysis in the replication set.

For IA genes from the literature, we listed the p-values of differential expression in the discovery set (S1 Table). In total, 87 probes that mapped to these 69 IA genes were tested in our study. No probes were differentially expressed between patients and controls with p_Bonf_ < 0.05.

### 2. Co-differential co-expression

We identified nine different modules of co-expressed genes in the discovery set of cases and controls combined. In none of these modules, the average gene significance level of differential expression of the genes was significantly higher than expected by chance, with p-values ranging from 0.23 to 0.48.

### 3. Differential co-expression and biological relevance of gene modules

After creating a dendrogram of DC genes in the discovery set, we observed six different modules of DC genes (S2 Fig). After 10 000 permutations, three modules remained significant (Table 2): the blue (p_Bonf_ = 0.024), turquoise (p_Bonf_ = 0.045) and yellow module (p_Bonf_ = 0.047).

**Table 2:** Modules of differentially co-expressed genes: permutation and preservation results.

Module	Number of genes	Permutation index	Permutation p_Bonf_	Preservation Zsummary
Blue	2007	40	0.024	6.06
Black	2837	553	0.33	NA
Brown	1822	103	0.062	NA
Green	1598	329	0.20	NA
Turquoise	3106	75	0.045	-0.74
Yellow	1818	81	0.047	11.32

p_Bonf_ indicates Bonferroni-corrected p-value, NA: not applicable (module not tested for preservation). Modules were randomly color-labeled.

The correlation heatmaps of differentially co-expressed gene modules showed that the pattern of case-control differences in gene correlation per module, as observed in the discovery set, did not look similar in the replication set (S3 Fig). We formally tested the preservation of the three significant modules in the replication set, and observed a significant preservation of only the yellow module (Z-summary = 11.3; Table 2). This module consisted of 1818 probes. Pathway analysis in DAVID revealed that only Gene Ontology pathways involved in processes in the vacuole and lysosome were significantly enriched in this module (p_Bonf_ = 5 x 10^−3^). We did not find any publications on a role of such processes in IA.

We identified a total number of 129 genes from the yellow module as hub genes. No overlap was found between the 69 IA genes and the list of hub genes. The most important hub gene of the yellow module was *CLCN6* (chloride channel, voltage-sensitive 6) at locus 1p36.22. This gene encodes a member of the voltage-dependent chloride channel protein family. An association with diastolic blood pressure has been described for a polymorphism in the promoter region of this gene.[29] For the remaining genes in the ‘top 10’ of hub genes, no functions or disease associations with a known relation to IA were found. The ten most important hub genes are described in Table 3.

**Table 3:** Top 10 hub genes in yellow module.

Rank	Gene	Locus	Product / function	Disease associations
1	*CLCN6*	1p36	Voltage-gated chloride channel proteins	Blood pressure
2	*SAG*	2q37	Cellular responses in retina and pineal gland	Oguchi disease
3	*AOC2*	17q21	Copper binding	Ocular diseases
4	*LILRA6*	19q13	Leukocyte immunoglobulin-like receptor	-
5	*PCDHA5*	5q31	Neural cell adhesion proteins	-
6	*LYPD6*	2q23	Disulfide-bonding proteins	-
7	*LHX6*	9q33	Development of neural and lymphoid cells	Lung cancer, cervical cancer
8	*LOC390531*	15q11	Non-functional	-
9	*CISH*	3p21	Regulation of cytokine-signaling	Infectious diseases
10	*HNMT*	2q22	Metabolization of histamine	Parkinson’s disease

### 4. Prediction analysis of microarrays (PAM)

**Fig 1:**
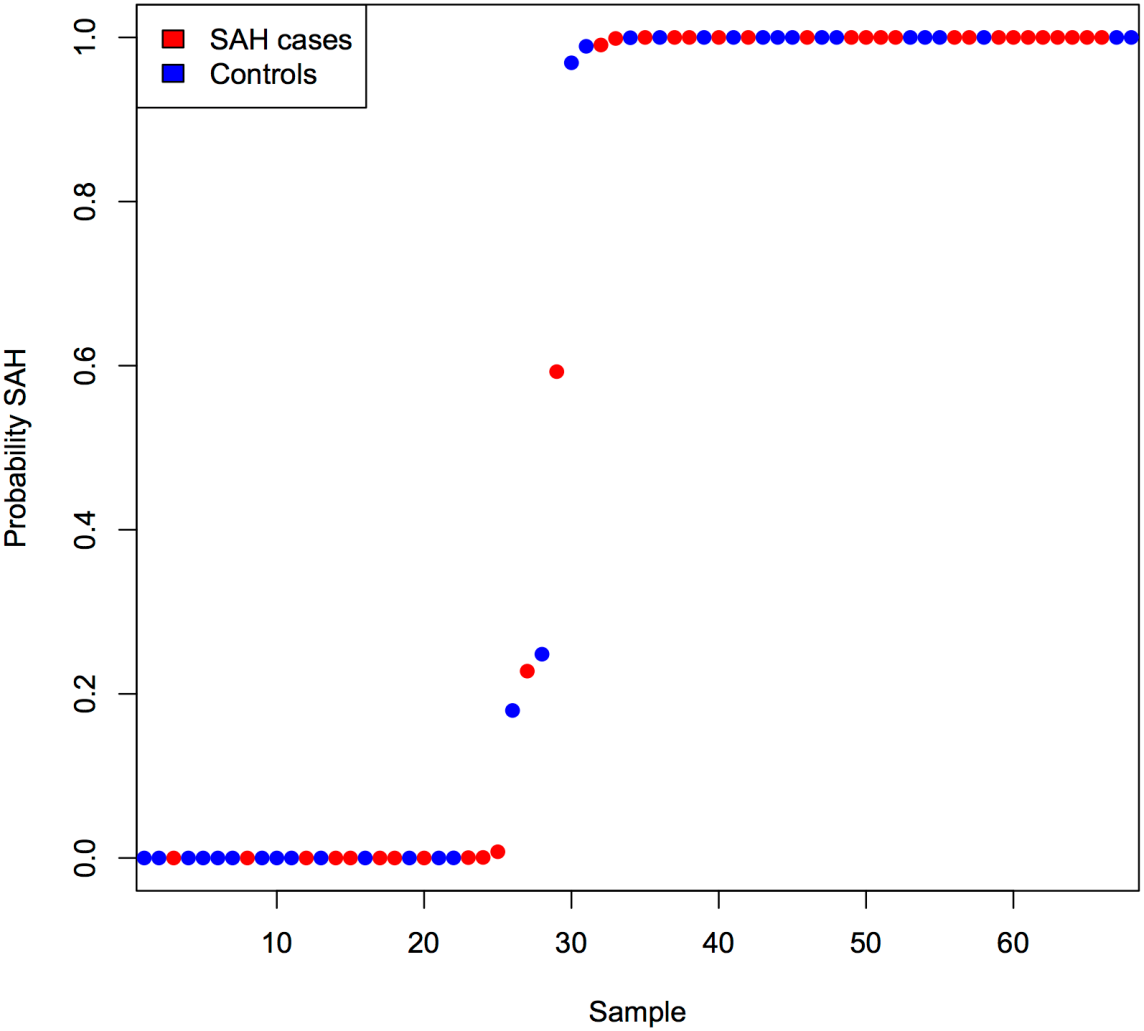
Predicted SAH probability in subjects from replication set, using 2388 probes selected with prediction analysis of microarrays. This figure shows the probability of being a SAH case for each subject in the replication set, based on prediction analysis of microarrays (PAM). We used PAM to define a group of probes in the discovery set with the highest predictive value to identify cases and controls in the replication set. As a result, a group of 2388 probes was selected, with a relatively high misclassification rate of 40%. The figure shows that this group of probes does not divide cases and controls in two separate groups.

The gene sets created with PAM in the discovery set all had a low predictive ability for classifying samples in the replication set as cases and controls, with misclassification rates of 40% or higher. The smallest gene set with a misclassification rate of 40% consisted of 2388 genes. This gene set did not cluster cases and controls in separate groups in the replication set (Fig 1), with a sensitivity for detecting SAH cases of 66%, and a specificity of 64%.

## Discussion

We found no significantly differentially expressed genes in peripheral blood between patients with a history of aSAH and controls. We identified only one group of genes with a significant and preservated case-control difference in co-expression, which can indicate the presence of coding changes in one or more genes present in that group. However, it remains unclear whether these genes are biologically relevant. First, we could not ascribe biological meaning either to this group of genes, or to the most connected genes within this network, the so-called ‘hub’ genes, according to pathway analysis. Specifically, the gene groups were not involved in pathophysiological processes known to be involved in IA formation and rupture, like vessel wall degeneration and inflammation.[4, 5] Second, these genes were not present in a list of 69 genes with a previously described association to IA based on GWAS[14–16] and gene expression studies.[17] In addition, we did not find a set of differentially expressed genes with the ability to predict disease status.

Previous studies on gene expression in IA mainly investigated gene expression in IA tissue, and some genes were differentially expressed between IA and control tissue in multiple studies: *BCL2*, *COL1A2*, *COL3A1*, *COL5A2*, *CXCL12*, *TIMP4*, and *TNC*.[17] In our study, these genes were not differentially expressed in blood (S1 Table) and did also not play an important role in DC. It should be noted that many of the previous IA tissue gene expression studies had limitations, in particular most were small. Moreover, their study designs were heterogeneous. These factors could limit the number of genes that were differentially expressed in multiple studies. One relatively large gene expression study in tissue of IA at the middle cerebral artery discovered upregulation of several biological processes in ruptured compared to unruptured IA: response to turbulent blood flow, chemotaxis, leukocyte migration, oxidative stress, vascular remodeling, and extracellular matrix degradation.[30] These processes were also not found in our pathway analysis. The most likely explanation for the discrepancies between the IA tissue studies and our study findings is that the published genes could be expressed specifically in IA tissue and not in blood. Additionally, some of these genes[30] could be specifically involved in IA rupture and not in IA development.

Our motivation to study gene expression in blood is the higher clinical relevance compared to IA tissue studies: because blood is accessible and easy to obtain, gene expression differences present in blood could potentially lead to development of a biomarker.

Recently, two studies on gene expression in blood of aSAH patients have been published. One study included aSAH patients in the acute phase and found expression differences of genes related to T lymphocytes, monocytes and neutrophils.[31] This study is not comparable to our study, in which we explicitly wanted to avoid including patients in the acute phase, since these acute consequences probably do not tell anything about the pathogenesis of development and rupture of IA. In a second study, in which gene expression in blood was compared between 30 patients with ruptured and unruptured IA and 15 controls, expression differences were found for genes coding for matrix metalloproteinases, extracellular matrix and cytoskeleton proteins and of genes related to apoptosis.[32] However, no correction for multiple testing and no replication experiments were performed. The timing of blood sampling in relation to aSAH (in case of ruptured IA) and to treatment of the IA is not described, thus we cannot tell whether the differences found are secondary to rupture or treatment, or are related to IA development.

Gene expression profiling has already been proven to characterize disease status in several vascular diseases, including transient ischemic attacks (TIAs)[33] and ischemic stroke.[34] Gene expression studies in blood have also been performed for abdominal aortic aneurysms (AAA)[35] and thoracic aortic aneurysms (TAA).[36] In contrast to the present study, these studies did reveal gene expression differences. In AAA, some disease-specific gene expression differences were found both in blood and in tissue, while in TAA it was even possible to predict disease state with gene expression profiles. However, a direct comparison with our study is not straightforward, because it is not clearly stated for these studies in which stage of the disease the blood samples were taken (before or after aneurysm rupture, and before or after treatment). Secondary effects of aneurysm rupture and operative treatment on gene expression are therefore not excluded.

A strength of this study is the fact that we obtained blood samples several years after aSAH. In this way, we minimized secondary effects of IA rupture on gene expression. To our knowledge, the sample size of this study is the largest compared to previous gene expression studies in blood of aneurysms and of other cardiovascular diseases.[37] Furthermore, to minimise confounding we matched cases and controls for factors including age and lifestyle by including mainly spouses of aSAH patients as controls, and blood samples were obtained at the same time and under the same circumstances for all participants. We also adjusted for known risk factors of IA and aSAH. In this way, we minimized influence of factors not related to the disease, with a known influence on gene expression.[13]

Our retrospective study design, using previous aSAH patients, could lead to limitations. First, disease-specific gene expression differences can theoretically be undetectable after IA treatment. Ideally, we would also have studied patients with unruptured IA who are followed-up for growth of their IA, using growth as a surrogate risk factor of rupture,[38] and compare gene expression between IA that grow to those IA that remain stable over time. However, such a study is difficult to perform given the low availability of such patients. Also, the usage of previous aSAH patients to study IA pathogenesis can be justified, as IA appears to be a continuous disease process in the intracranial vessel wall: previous aSAH patients are still at risk of developing new aneurysms, and of growth of already present unruptured IA.[10]

Second, the study design precludes patients who deceased after aSAH. This selection bias could theoretically have influenced our results, but it is unlikely that disease-specific gene expression differences are only detectable in patients with a poor outcome.

In conclusion, this study revealed no structural gene expression differences in blood of previous aSAH patients compared to controls. Also, gene expression profiles in blood of previous aSAH patients, as detected by expression arrays, do not seem promising for development of clinically useful biomarkers. Future studies could aim at detecting changes in gene expression profiles in patients with yet unruptured IA that grow over time, or at the identification of other potential disease markers, including microRNAs or using proteomics.

## Supporting Information

S1 Fig Principal component (PC) plot of cases and controls. This figure shows the first two PCs (two independent variables explaining most of the data variance) from the raw gene expression dataset, plotted against each other. Cases are depicted in red and controls are depicted in blue. The nine cases and controls left from the dashed line were identified as outliers, based on visual inspection. The figure further shows that cases and controls do not cluster in separate groups based on these PCs.
(TIF)

S2 Fig Dendrogram and gene clustering of differentially co-expressed genes in the discovery set. We applied hierarchical clustering on a differential co-expression matrix: a matrix of case control differences in pair-wise correlations between all genes. This is visualized in the dendrogram above. Each line represents a gene, and the y-axis shows the level of differential co-expression between genes. Low branches mean high case-control differences in gene correlation. Based on these branches, we created six clusters of differential co-expressed genes (a black, green, brown, turquoise, yellow, and blue module), using a tree cut height of 0.96.[21]
(TIF)

S3 Fig Correlation heatmaps of differentially co-expressed gene modules. Correlation heatmaps of differential co-expressed genes in cases (upper diagonal) compared to controls (lower diagonal). Positive correlations are shown in red, negative correlations in blue. In the discovery set (A), the gene correlations in most modules appear to be different between cases and controls. When looking at the same gene correlations in the replication set (B), a different pattern of correlation differences is visible. The right part of the figure shows that the mean gene expression level of all modules is low, both in cases and controls.
(TIF)

S1 Table List of known intracranial aneurysm genes from previous genetic studies, with differential expression results for each corresponding probe. (DOCX)

S2 Table Leukocyte differential counts in cases versus controls. (DOCX)
