# Supplementary figures and images for: Whole Blood Gene Expression Profiles of Patients with a Past Aneurysmal Subarachnoid Hemorrhage
Source: PLoS One. 2015 Oct 6;10(10):e0139352. doi: 10.1371/journal.pone.0139352 (PMC4595144; doi:10.1371/journal.pone.0139352)

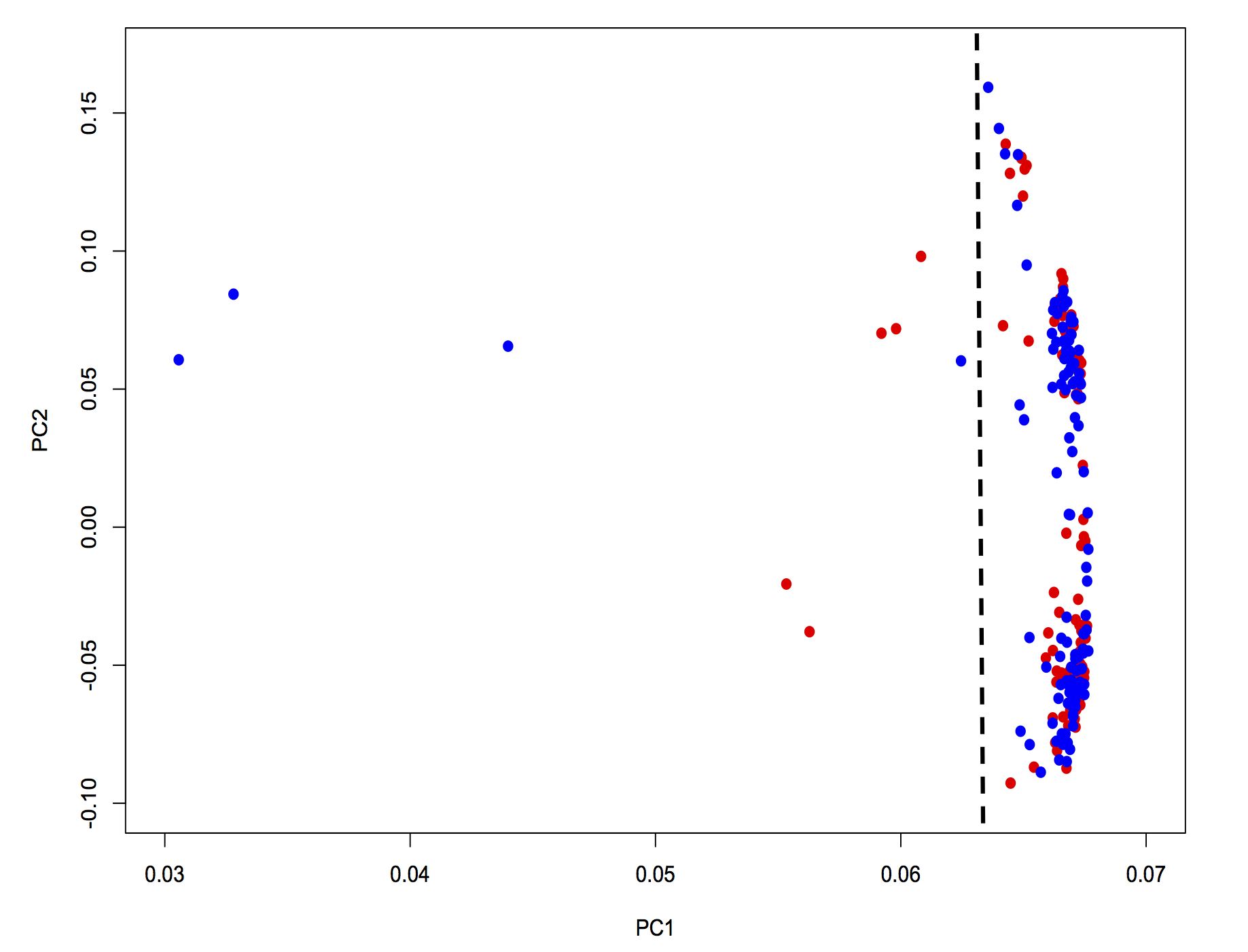

Supplement: S1 Fig — This figure shows the first two PCs (two independent variables explaining most of the data variance) from the raw gene expression dataset, plotted against each other. Cases are depicted in red and controls are depicted in blue. The nine cases and controls left from the dashed line were identified as outliers, based on visual inspection. The figure further shows that cases and controls do not cluster in separate groups based on these PCs. (TIF) [file pone.0139352.s001.tif]

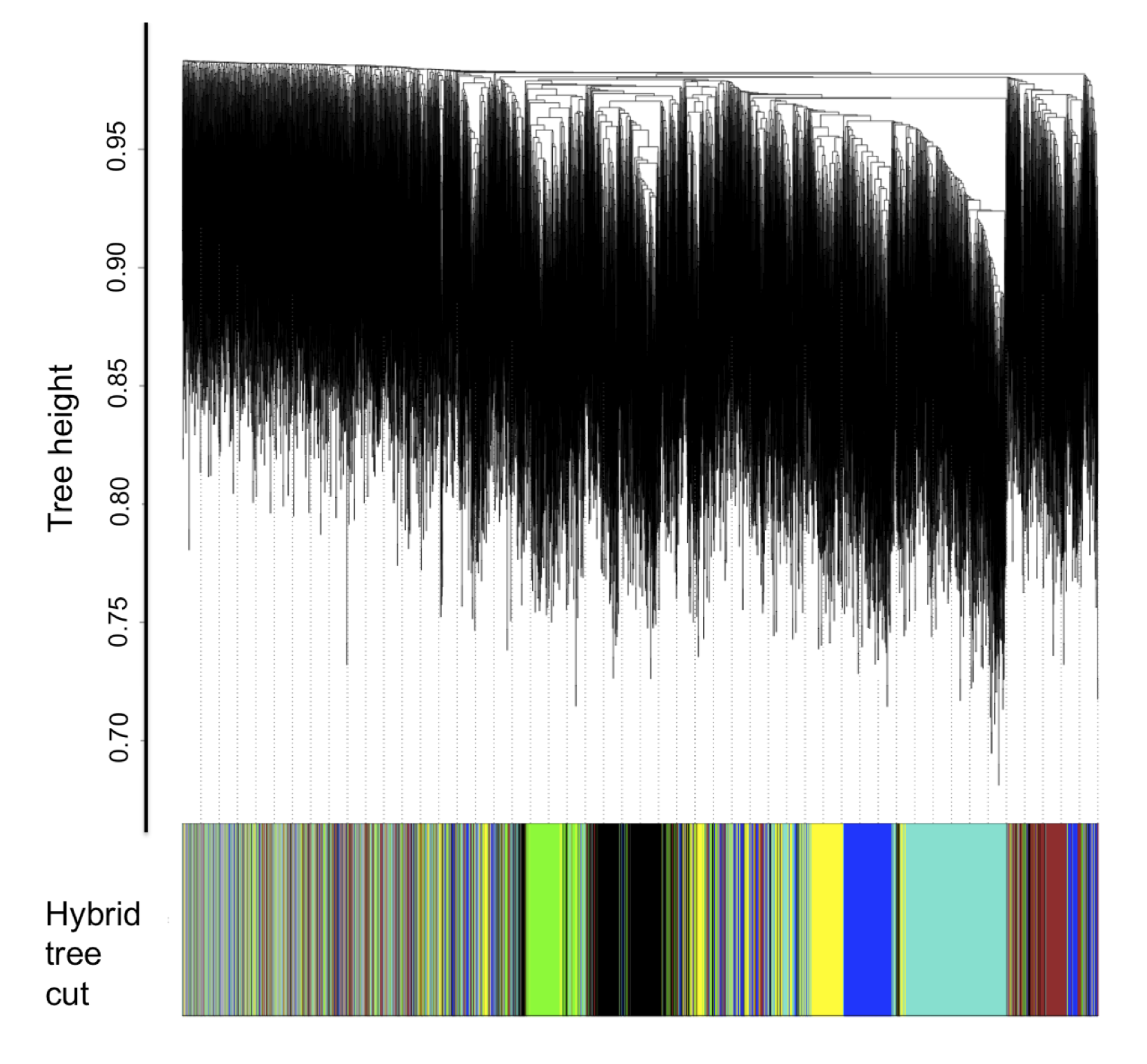

Supplement: S2 Fig — We applied hierarchical clustering on a differential co-expression matrix: a matrix of case control differences in pair-wise correlations between all genes. This is visualized in the dendrogram above. Each line represents a gene, and the y-axis shows the level of differential co-expression between genes. Low branches mean high case-control differences in gene correlation. Based on these branches, we created six clusters of differential co-expressed genes (a black, green, brown, turquoise, yellow, and blue module), using a tree cut height of 0.96.[21] (TIF) [file pone.0139352.s002.tif]

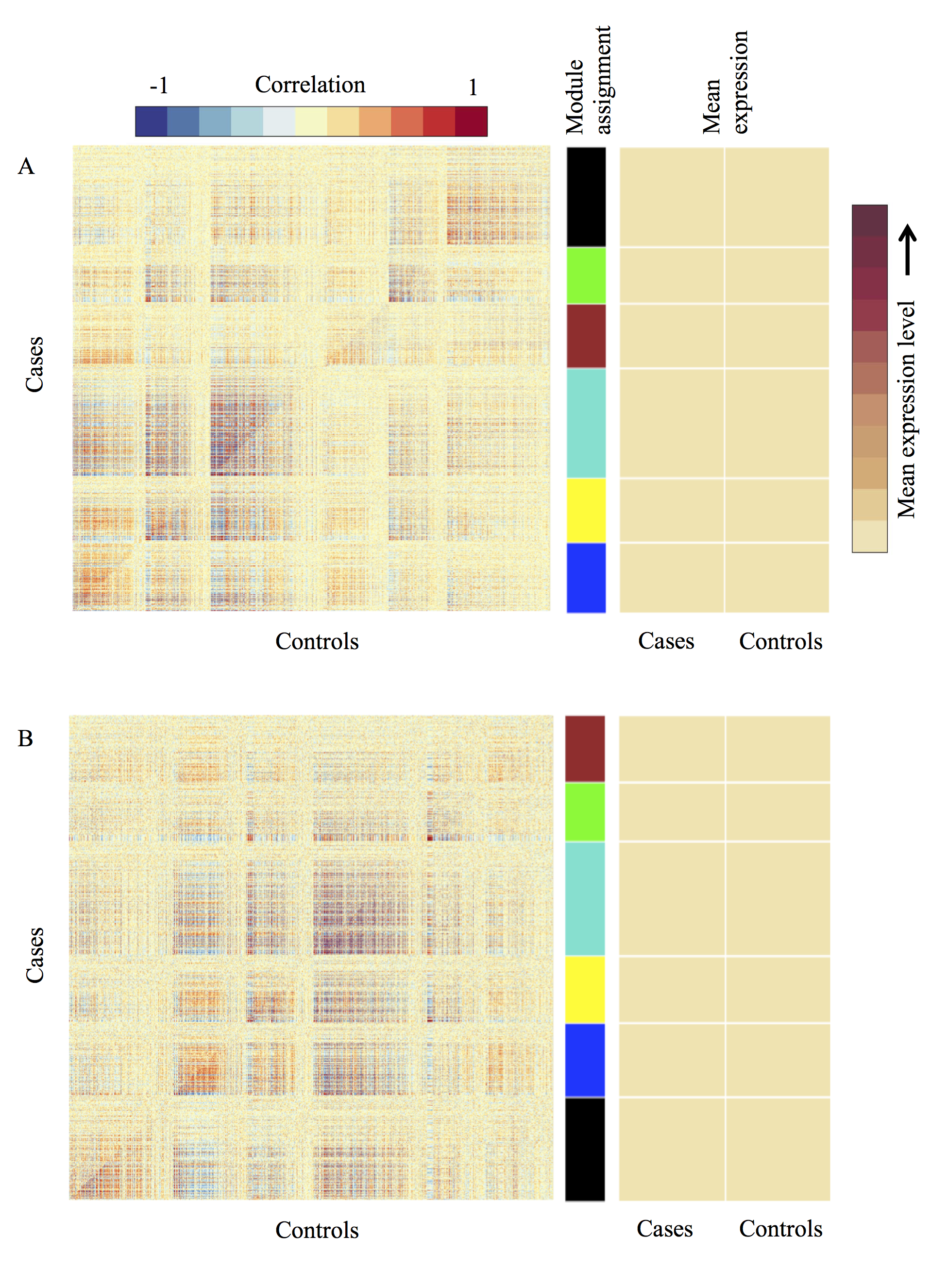

Supplement: S3 Fig — Correlation heatmaps of differential co-expressed genes in cases (upper diagonal) compared to controls (lower diagonal). Positive correlations are shown in red, negative correlations in blue. In the discovery set (A), the gene correlations in most modules appear to be different between cases and controls. When looking at the same gene correlations in the replication set (B), a different pattern of correlation differences is visible. The right part of the figure shows that the mean gene expression level of all modules is low, both in cases and controls. (TIF) [file pone.0139352.s003.tif]
