# Supplementary material for: Whole Blood Gene Expression Profiles of Patients with a Past Aneurysmal Subarachnoid Hemorrhage
Source: PLoS One. 2015 Oct 6;10(10):e0139352. doi: 10.1371/journal.pone.0139352 (PMC4595144; doi:10.1371/journal.pone.0139352)
Supplement: S1 Table — (DOCX) [file pone.0139352.s004.docx]

**Table S1:** List of known intracranial aneurysm genes from previous genetic studies, with differential expression results for each corresponding probe.

| **Gene** | **Locus** | **Reference** |  | **Probe ID** | **p-value** | **Beta** |
| --- | --- | --- | --- | --- | --- | --- |
| SOX17 | 8q11-q12 | [1](#_ENREF_1) |  | 3610193 | 0.184 | 0.015 |
| RP1 | 8q11-q12 | [1](#_ENREF_1) |  | 3130040 | 0.477 | 0.011 |
| " |  |  |  | 2630326 | 0.003 | -0.051 |
| PDS5B | 13q13 | [1](#_ENREF_1) |  | 5220575 | 0.580 | -0.009 |
| " |  |  |  | 870167 | 0.314 | -0.017 |
| KL | 13q13 | [1](#_ENREF_1) |  | 1190392 | 0.629 | -0.005 |
| STARD13 | 13q13 | [1](#_ENREF_1) |  | 2570255 | 0.171 | -0.025 |
| " |  |  |  | 3190411 | 0.932 | 0.001 |
| " |  |  |  | 290270 | 0.642 | 0.005 |
| " |  |  |  | 6060632 | 0.451 | -0.013 |
| " |  |  |  | 3440184 | 0.233 | -0.021 |
| MTAP | 9p21 | [1](#_ENREF_1) |  | 2600291 | 0.857 | 0.003 |
| CDKN2A | 9p21 | [1](#_ENREF_1) |  | 5130671 | 0.642 | 0.006 |
| " |  |  |  | 5550671 | 0.923 | 0.001 |
| " |  |  |  | 6620014 | 0.981 | 0.000 |
| CDKN2B | 9p21 | [1](#_ENREF_1) |  | 1500338 | 0.084 | -0.025 |
| " |  |  |  | 7610735 | 0.953 | -0.001 |
| " |  |  |  | 620487 | 0.677 | 0.009 |
| CTAGE1 | 18q11 | [1](#_ENREF_1) |  | 5090427 | 0.667 | -0.006 |
| " |  |  |  | 2710504 | 0.160 | -0.014 |
| RBBP8 | 18q11 | [1](#_ENREF_1) |  | 6580672 | 0.492 | -0.009 |
| " |  |  |  | 6060138 | 0.450 | -0.012 |
| " |  |  |  | 5720133 | 0.287 | -0.015 |
| CABLES1 | 18q11 | [1](#_ENREF_1) |  | 4050411 | 0.235 | 0.016 |
| C18ORF45 | 18q11 | [1](#_ENREF_1) |  | NA | NA | NA |
| TMEM241 | 18q11 | [1](#_ENREF_1) |  | NA | NA | NA |
| RIOK3 | 18q11 | [1](#_ENREF_1) |  | 360187 | 0.500 | -0.023 |
| " |  |  |  | 4210019 | 0.418 | -0.032 |
| " |  |  |  | 360187 | 0.500 | -0.023 |
| C18ORF8 | 18q11 | [1](#_ENREF_1) |  | NA | NA | NA |
| NPC1 | 18q11 | [1](#_ENREF_1) |  | 7570358 | 0.212 | 0.021 |
| ANKRD29 | 18q11 | [1](#_ENREF_1) |  | 3130114 | 0.653 | -0.008 |
| " |  |  |  | 1500358 | 0.794 | -0.003 |
| LAMA3 | 18q11 | [1](#_ENREF_1) |  | 4120358 | 0.842 | -0.003 |
| " |  |  |  | 4670553 | 0.045 | -0.029 |
| " |  |  |  | 2650612 | 0.043 | -0.030 |
| NOLC1 | 10q24 | [1](#_ENREF_1) |  | 5960132 | 0.518 | 0.009 |
| ELOVL3 | 10q24 | [1](#_ENREF_1) |  | 5260056 | 0.953 | 0.001 |
| PITX3 | 10q24 | [1](#_ENREF_1) |  | 4200564 | 0.251 | -0.020 |
| GBF1 | 10q24 | [1](#_ENREF_1) |  | 6510192 | 0.001 | -0.061 |
| NFKB2 | 10q24 | [1](#_ENREF_1) |  | 3310615 | 0.051 | -0.049 |
| " |  |  |  | 3830239 | 0.830 | -0.003 |
| PSD | 10q24 | [1](#_ENREF_1) |  | 2190543 | 0.768 | 0.005 |
| FBXL15 | 10q24 | [1](#_ENREF_1) |  | 7160364 | 0.731 | 0.007 |
| CUEDC2 | 10q24 | [1](#_ENREF_1) |  | 7040037 | 0.313 | 0.025 |
| C10ORF95 | 10q24 | [1](#_ENREF_1) |  | NA | NA | NA |
| TMEM180 | 10q24 | [1](#_ENREF_1) |  | 3870193 | 0.785 | -0.005 |
| ACTR1A | 10q24 | [1](#_ENREF_1) |  | NA | NA | NA |
| SUFU | 10q24 | [1](#_ENREF_1) |  | 2710397 | 0.268 | 0.016 |
| TRIM8 | 10q24 | [1](#_ENREF_1) |  | 940435 | 0.268 | 0.029 |
| ARL3 | 10q24 | [1](#_ENREF_1) |  | 7150671 | 0.825 | -0.003 |
| SFXN2 | 10q24 | [1](#_ENREF_1) |  | 5720553 | 0.284 | 0.013 |
| C10ORF26 | 10q24 | [1](#_ENREF_1) |  | NA | NA | NA |
| WBP1L | 10q24 | [1](#_ENREF_1) |  | NA | NA | NA |
| CYP17A1 | 10q24 | [1](#_ENREF_1) |  | 3890278 | 0.883 | -0.002 |
| C10ORF32 | 10q24 | [1](#_ENREF_1) |  | NA | NA | NA |
| AS3MT | 10q24 | [1](#_ENREF_1) |  | 4280390 | 0.220 | -0.017 |
| CNNM2 | 10q24 | [1](#_ENREF_1) |  | 4060730 | 0.555 | 0.009 |
| " |  |  |  | 6620184 | 0.859 | 0.002 |
| " |  |  |  | 1070605 | 0.479 | -0.010 |
| NT5C2 | 10q24 | [1](#_ENREF_1) |  | 1010253 | 0.251 | -0.031 |
| INA | 10q24 | [1](#_ENREF_1) |  | 1230538 | 0.636 | -0.006 |
| PCGF6 | 10q24 | [1](#_ENREF_1) |  | NA | NA | NA |
| TAF5 | 10q24 | [1](#_ENREF_1) |  | 2900193 | 0.555 | 0.008 |
| USMG5 | 10q24 | [1](#_ENREF_1) |  | 2320253 | 0.262 | -0.022 |
| PDCD11 | 10q24 | [1](#_ENREF_1) |  | NA | NA | NA |
| CALHM2 | 10q24 | [1](#_ENREF_1) |  | 1440241 | 0.471 | -0.024 |
| CALHM1 | 10q24 | [1](#_ENREF_1) |  | NA | NA | NA |
| CALHM3 | 10q24 | [1](#_ENREF_1) |  | NA | NA | NA |
| NEURL | 10q24 | [1](#_ENREF_1) |  | 110458 | 0.464 | 0.010 |
| SH3PXD2A | 10q24 | [1](#_ENREF_1) |  | 3460451 | 0.418 | 0.015 |
| EDNRA | 4q31 | [2](#_ENREF_2) |  | 3930403 | 0.958 | 0.001 |
| TMEM184C | 4q31 | [2](#_ENREF_2) |  | NA | NA | NA |
| LOC90826 | 4q31 | [2](#_ENREF_2) |  | NA | NA | NA |
| PRMT10 | 4q31 | [2](#_ENREF_2) |  | 2450553 | 0.149 | -0.029 |
| ARHGAP10 | 4q31 | [2](#_ENREF_2) |  | 2000669 | 0.925 | -0.002 |
| NDUFA12 | 12q22 | [2](#_ENREF_2) |  | 5090050 | 0.408 | 0.028 |
| NR2C1 | 12q22 | [2](#_ENREF_2) |  | 1470521 | 0.985 | 0.000 |
| " |  |  |  | 270544 | 0.828 | 0.004 |
| FGD6 | 12q22 | [2](#_ENREF_2) |  | NA | NA | NA |
| VEZT | 12q22 | [2](#_ENREF_2) |  | 2000195 | 0.471 | 0.018 |
| " |  |  |  | 5550475 | 0.439 | -0.014 |
| BFSP1 | 20p12 | [2](#_ENREF_2) |  | 2370364 | 0.520 | -0.010 |
| DSTN | 20p12 | [2](#_ENREF_2) |  | 6900612 | 0.172 | 0.041 |
| " |  |  |  | 3190634 | 0.059 | 0.068 |
| " |  |  |  | 4590470 | 0.805 | -0.005 |
| RRBP1 | 20p12 | [2](#_ENREF_2) |  | 3890408 | 0.851 | -0.005 |
| " |  |  |  | 1230307 | 0.016 | -0.042 |
| " |  |  |  | 4540327 | 0.108 | -0.034 |
| BANF2 | 20p12 | [2](#_ENREF_2) |  | 1570072 | 0.369 | -0.016 |
| BCL2 | 18q21 | [3](#_ENREF_3) |  | 3180494 | 0.087 | 0.049 |
| " |  |  |  | 4150201 | 0.021 | 0.067 |
| COL1A2 | 7q22 | [3](#_ENREF_3) |  | 6060612 | 0.791 | -0.004 |
| " |  |  |  | 4040671 | 0.428 | -0.014 |
| COL3A1 | 2q31 | [3](#_ENREF_3) |  | 2120021 | 0.131 | -0.024 |
| COL5A2 | 2q14-q32 | [3](#_ENREF_3) |  | 4490292 | 0.778 | -0.003 |
| CXCL12 | 10q11 | [3](#_ENREF_3) |  | 4610615 | 0.771 | 0.003 |
| " |  |  |  | 4560020 | 0.255 | -0.016 |
| " |  |  |  | 3870253 | 0.933 | -0.001 |
| TIMP4 | 3p25 | [3](#_ENREF_3) |  | 1090326 | 0.494 | 0.009 |
| TNC | 9q33 | [3](#_ENREF_3) |  | 1470669 | 0.982 | 0.000 |
| " |  |  |  | 6940102 | 0.828 | -0.006 |

*NA indicates not available (no probe available for this gene in our study results).*

References

1. Yasuno K, Bilguvar K, Bijlenga P, Low SK, Krischek B, Auburger G, et al. Genome-wide association study of intracranial aneurysm identifies three new risk loci. *Nat Genet*. 2010;42:420-425

2. Yasuno K, Bakircioglu M, Low SK, Bilguvar K, Gaal E, Ruigrok YM, et al. Common variant near the endothelin receptor type A (EDNRA) gene is associated with intracranial aneurysm risk. *Proc Natl Acad Sci U S A*. 2011;108:19707-19712

3. Roder C, Kasuya H, Harati A, Tatagiba M, Inoue I, Krischek B. Meta-analysis of microarray gene expression studies on intracranial aneurysms. *Neuroscience*. 2012;201:105-113
