# Supplementary material for: Whole Blood Gene Expression Profiles of Patients with a Past Aneurysmal Subarachnoid Hemorrhage
Source: PLoS One. 2015 Oct 6;10(10):e0139352. doi: 10.1371/journal.pone.0139352 (PMC4595144; doi:10.1371/journal.pone.0139352)
Supplement: S2 Table — (DOCX) [file pone.0139352.s005.docx]

**Table S2:** Leukocyte differential counts in cases versus controls.

| Cell type | Cases:  Mean (SD) | Controls:  Mean (SD) | p-value |
| --- | --- | --- | --- |
| Leukocytes  Eosinophils**^*^** | 6.94 (1.89)  3.04 (2.04) | 6.59 (2.03)  2.98 (1.75) | 0.06  0.85 |
| Basophils**^*^**  Neutrophils**^*^**  Lymphocytes**^*^**  Monocytes**^*^** | 0.81 (0.51)  57.0 (8.70)  30.94 (7.45)  8.17 (2.41) | 0.74 (0.44)  54.74 (11.57)  32.71 (8.28)  8.28 (2.67) | 0.34  0.08  0.06  0.55 |

***^*^*** *Percentage of absolute leukocyte count*
